# NGS Analysis of Clonality and Minimal Residual Disease in a Patient with Concurrent Richter's Transformation and CLL/SLL

**DOI:** 10.1155/2021/9740281

**Published:** 2021-12-28

**Authors:** Shrihari S. Kadkol, Joshua Bland, Ashley Kavanaugh, Hongyu Ni, Vijeyaluxmi Nehru, David Peace

**Affiliations:** ^1^Department of Pathology, University of Illinois, Chicago, Chicago, IL, USA; ^2^Department of Medicine, University of Illinois at Chicago, Chicago, IL, USA

## Abstract

B-cell lymphomas are neoplastic proliferations of clonal B lymphocytes. Clonality is generally determined by PCR amplification of VDJ rearrangements in the IgH heavy chain or VJ rearrangements in Ig*κ*/Ig*λ* light chain genes followed by capillary electrophoresis. More recently, next-generation sequencing (NGS) has been used to detect clonality in B-cell lymphomas because of the exponential amount of information that is obtained beyond just detecting a clonal population. The additional information obtained is useful for diagnostic confirmation, prognosis assessment, and response to therapy. In this study, we utilized NGS analysis to characterize two histologically distinct lymphomas (DLBCL and CLL/SLL) that were detected contemporaneously in an asymptomatic patient. NGS analysis showed that the same VDJ rearrangement was present in nodal (DLBCL) and marrow (CLL/SLL) biopsies confirming that the DLBCL resulted from Richter's transformation of a subclinical CLL/SLL. The V region of the rearrangement remained unmutated without somatic hypermutation. In silico analysis showed that the HCDR3 sequence was heterogeneous and not stereotypic. Minimal residual disease analysis by NGS showed that the tumor clone decreased by 2.84 logs in the bone marrow after R-CHOP therapy. However, a small number of tumor cells were still detected in the peripheral blood after R-CHOP therapy. Subsequent allogeneic transplantation was successful in eradicating the tumor clone and achieving deep molecular remission. We show that NGS analysis facilitated clinical management in our patient by helping to characterize the VDJ rearrangement in detail and by tracking minimal residual disease with high sensitivity and specificity.

## 1. Introduction

B-cell lymphomas are characterized by clonal expansion of B lymphocytes that contain specific VDJ or VJ rearrangements in IgH and Ig*κ*/Ig*λ* genes. There are approximately 50–60 functional *V*, 25 *D*, and 6 J segments on the IgH locus on chromosome 14 [1]. D-J followed by *V*-DJ joining results in the first level of heavy-chain diversity. This is followed by random nucleotide additions and/or deletions at the D-J and V-DJ junctions that result in the next level of diversity. Many of the rearrangements are unsuccessful and do not result in a functional IgH chain due to premature stop codons or frameshifts. If the rearrangement is unsuccessful on one allele, the B cell attempts rearrangement on the second IgH allele on chromosome 14. If one of the rearrangements in the IgH locus is successful, rearrangement proceeds to the light chains. In a successful VDJ rearrangement on the IgH gene, the 3′ end of *V* and *D* and 5′ end of the *J* segment code for the HCDR3 region of the heavy chain. As a result of nucleotide addition and deletion in the junctional regions, the HCDR3 region contributes maximally towards antibody diversity.

VDJ rearrangements can be detected by PCR amplification with primers that bind the V and *J* segments [2]. Subsequently, the amplicons are analyzed by capillary electrophoresis to determine clonality [3]. A rearrangement of a particular length that is overrepresented is suggestive of a clonal population. However, capillary electrophoresis resolves amplicons based on length alone. In contrast, VDJ amplicon sequencing by NGS has distinct advantages over just length separation and include the ability to determine the (i) identity of the *V*, *D*, and *J* segments, (ii) clonal relationship between histologically distinct lymphomas, (iii) somatic hypermutation (SHM) in the *V* region, (iv) B-cell receptor stereotypy based on HCDR3 length and composition, and (v) minimal residual disease (MRD) with high sensitivity. The ability to obtain multiple pieces of information with a single assay is particularly advantageous for optimizing clinical management in patients with B-cell lymphomas.

Recently, NGS has enabled the analysis of VDJ amplicons at an unprecedented level of depth with implications for diagnosis, prognosis, and therapy [4]. In this case report, we highlight the role of NGS analysis in confirming Richter's transformation of CLL/SLL to diffuse large B-cell lymphoma (DLBCL) in an asymptomatic patient. We demonstrate the power of NGS to characterize the clonal VDJ rearrangement in detail and to follow minimal residual disease (MRD) with exquisite sensitivity in peripheral blood and bone marrow with implications for prognosis and therapy.

## 2. Case Presentation

**Figure 1:**
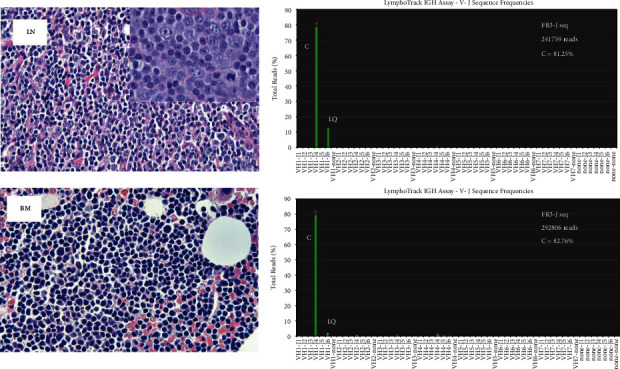
Tumor histology and V-J frequency graphs. On the left are H&E sections from the DLBCL in the lymph node and CLL/SLL in the bone marrow. Inset: high-power view of the DLBCL in the lymph node. On the right are the *V*-J frequency graphs from FR3-J primers showing the clonal and LQ reads. The *Y*-axis is the % of total reads, and *X*-axis is the specific *V*-J rearrangement. Clonal reads are present in both the lymph node and bone marrow. C: clonal, V1-2^*∗*^02/*D*5-12^*∗*^01/J4^*∗*^02, LQ: LymphoQuant control, and V1-69^*∗*^01/*D*6-13^*∗*^01/J6^*∗*^03.

A 55-year-old woman presented with mild enlargement of a right posterior cervical node over a period of several months. She had an excellent performance status and denied any constitutional complaints. Excisional biopsy of the enlarged node demonstrated diffuse large B-cell lymphoma (Figure 1, LN, Supplementary File 2). H&E sections showed diffuse effacement of normal architecture by a lymphocytic infiltrate, consisting of sheets of large atypical cells in a background of small lymphocytes. The large atypical lymphocytes contained a small amount of cytoplasm, oval to round nuclei, vesicular chromatin, and one to three prominent nucleoli. The Ki-67 positivity was around 40%, a value much higher than generally observed in CLL/SLL and consistent with DLBCL [5]. Based on these findings, the histopathology in the lymph node was diagnosed as DLBCL. The immunophenotype suggested a possible origin from an underlying CLL/SLL consistent with Richter's transformation (CD20+, CD23+, CD5+, BCL2+, BCL6−, CD10−, and cyclin D1−). The patient's complete blood count and metabolic profile were normal with an absolute lymphocyte count of 3.6 × 10^3^/*μ*l and serum LDH of 174 U/L (ULN 180). A CT-PET scan demonstrated bilateral FDG avid lymph nodes in the cervical, supraclavicular, and axillary regions. A staging bone marrow biopsy showed extensive involvement by small atypical lymphocytes with a kappa-restricted immunophenotype consistent with CLL/SLL (CD19+, CD20+, CD5+, CD23+, CD10−, and cyclin D1−). In contrast to the lymph node, there was no morphologic evidence of involvement by DLBCL in the bone marrow (Figure 1, BM). Cytogenetic analysis of the marrow aspirate showed a mosaic abnormal female karyotype with 40% of cells harboring a trisomy 12 abnormality.

### 2.1. Lymph Node and Bone Marrow Analysis by NGS (Supplementary File 1)

First, we determined the clonal relationship between the DLBCL in the lymph node and CLL/SLL in the bone marrow by next-generation sequencing (NGS). DNA extracted from FFPE sections of the lymph node and fresh bone marrow aspirate was analyzed for clonality by the NGS Lymphotrack IgH FR1/FR2/FR3 assay according to the manufacturer's protocol (Invivoscribe Inc., Cat no. 7-121-0139, San Diego, CA). DNA was first amplified with FR1-J, FR2-J, and FR3-J primers. Subsequently, the amplicons were purified and sequenced on an Illumina Miseq machine. Prior to amplification, a LymphoQuant B-cell internal control DNA (Invivoscribe, LQ, Cat no. 7-121-0139, 50 cell equivalents/µl) was added to each DNA specimen as a process control. Meticulous attention was followed to avoid contamination at each step. IgH clonality analysis was performed by importing the fastq.gz files into Lymphotrack software v2.4.3 (Invivoscribe). Using the merged read summary, a clonal sequence was defined as a sequence with a read frequency of >2.5% and a frequency of >10X the background (generally the 3^rd^ sequence in the merged read summary) [4].

**Figure 2:**
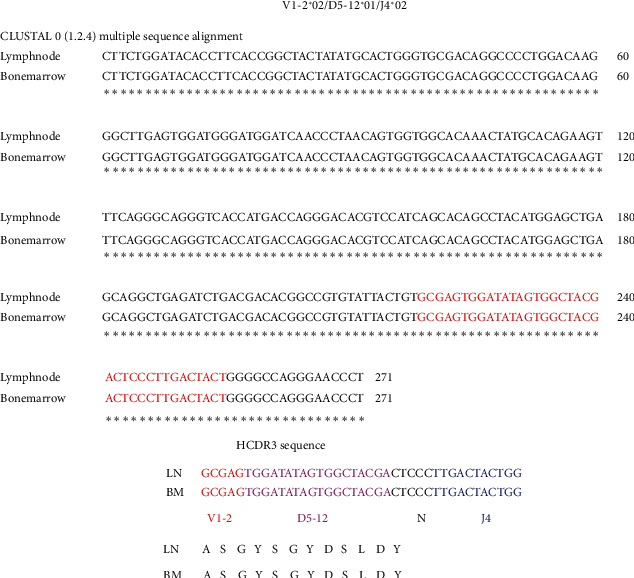
Alignment of the clonal sequence from lymph node and bone marrow. An identical clonal VDJ rearrangement is present in both specimens. The HCDR3 sequence is shown in red. The contribution of the *V*, *D*, and *J* segments to the HCDR3 sequence is shown (nucleotide, protein).

*V*-J frequency graphs after Lymphotrack analysis showed that the lymph node and bone marrow specimens from the patient contained a clonal sequence in FR1, FR2, and FR3 reactions. A representative FR3-J analysis with clonal reads is shown in Figure 1. Alignment analysis showed that the clonal rearrangement contained an identical sequence in both specimens, indicating that the same clone of B cells was present in both locations (Figure 2). IMGT V-Quest and IgBLAST analysis showed that the rearrangement was V1-2^*∗*^ 02/*D*5-12^*∗*^ 01/J4^*∗*^ 02. As expected, the sequence derived from the LQ process control was detected in both specimens (V1-69^*∗*^ 01/*D*6-13^*∗*^ 01/J6^*∗*^ 03) suggesting that performance of the assay was acceptable throughout all stages of the workflow. The total number of reads, % clonal (C), and % LQ sequence are shown in Table 1. A lower number of NGS reads were present in FR1-J and FR2-J reactions from the lymph node. FR1-J and FR2-J amplify lengths close to 350 bp and 250 bp, and because DNA is generally fragmented in FFPE tissue, a lower number of reads are to be expected. Nevertheless, the number of reads was still adequate to definitively call clonality because (i) review of the H&E sections showed almost complete effacement of the architecture by DLBCL ruling out a paucity of B cells as a cause of a false clonal call and (ii) FastQC analysis of the fastq.gz files showed that majority of the sequences were between 105 and 155 bp (200,000) with fewer sequences around 250 bp (∼100,000) reflecting the distribution of reads after Lymphotrack analysis. Furthermore, the per-base sequence quality was >*Q*30 in both paired reads until 250 bp indicating a high quality of the reads.

### 2.2. Detailed Characterization of the Clonal VDJ Rearrangement

**Figure 3:**
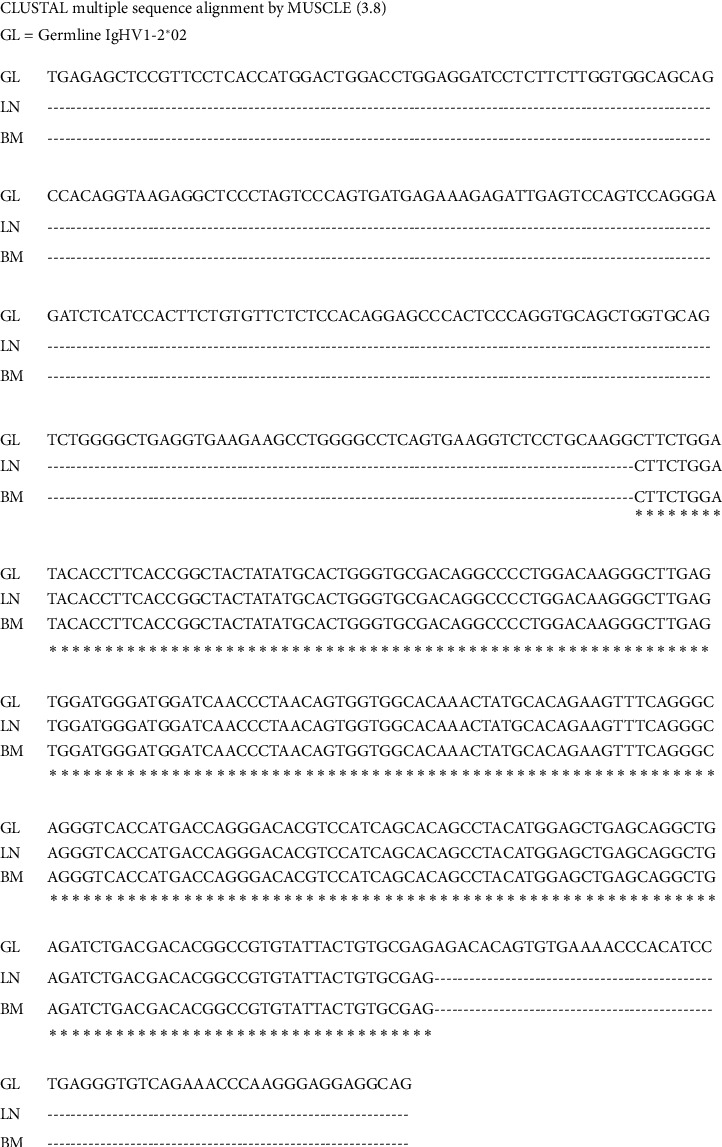
Alignment of the clonal VDJ sequence to germline V1-2^*∗*^02 The clonal sequence (FR1-J) from the lymph node and bone marrow shows an unmutated *V* region when aligned to the germline *V* gene. GL: germline sequence, LN: lymph node, and BM: bone marrow.

The same V1-2^*∗*^ 02/*D*5-12^*∗*^ 01/J4^*∗*^ 02 rearrangement was present in both lymph node and bone marrow specimens (Figure 2) confirming that the DLBCL in the lymph node arose as a result of Richter's transformation of a subclinical CLL/SLL. No other clonal sequence was identified indicating that the clone originated from a B cell with a monoallelic rearrangement. IMGT V-Quest and IgBLAST analysis showed that the rearrangement was productive. Alignment to the germline IGHV1-2^*∗*^ 02 sequence showed that the *V* region remained in an unmutated state suggesting an origin from a naïve B cell that had not yet experienced the germinal center reaction (Figure 3). The HCDR3 was 12 amino acids long: ASGYSGYDSLDY. The *V*-D junction did not show any additions or deletions. The D5-12^*∗*^01 segment was mostly intact except for the deletion of the last four nucleotides (GTGGATATAGTGGCTACGATTAC), but with an addition of five nucleotides (CTCCC) at the D-J junction.

### 2.3. Analysis of HCDR3 Stereotypy

It has been reported that the composition and length of the HCDR3 sequence may influence prognosis and risk of Richter's transformation [6–8]. Hence, we attempted to determine if the HCDR3 from the patient's sequence was stereotypic, because our patient seemed to have a rapid progression of CLL/SLL to DLBCL. Analysis of the FR1-J sequence by the web-based ARRest/AssignSubsets program (https://tools.bat.infspire.org/arrest/assignsubsets) showed that the patient's rearrangement was heterogeneous and did not fit into any of the 19 characterized subsets. Furthermore, the patient's HCDR3 protein sequence did not seem to align well by visual comparison with any of the reported 110 HCDR3 subsets [6,7]. As such, the patient's clonal HCDR3 sequence was considered to be heterogeneous.

### 2.4. Minimal Residual Disease Quantitation

Bone marrow and peripheral blood specimens were analyzed for minimal residual disease after the patient underwent 6 cycles of R-CHOP chemoimmunotherapy. A PET-CT scan at this time showed resolution of lymph node disease. Data were analyzed first by the Lymphotrack v2.4.3 software (Invivoscribe) and then by the LymphotrackMRD software v1.2.0 (Invivoscribe) to determine minimal residual disease. The clonal sequence that was unique to the tumor was tracked in follow-up specimens.

For each sample, the % clonal and % LQ sequence were calculated as follows:

  (Exact match clonal sequence reads/total reads) *×* 100 = % clonal sequence
  (LQ clonal sequence reads/total reads) *×* 100 = % LQ clonal sequence

MRD in cell equivalents/DNA input (ng) was calculated as follows:

  MRD = (% clonal sequence/% LQ clonal sequence) *×* 100 cell equivalents

**Figure 4:**
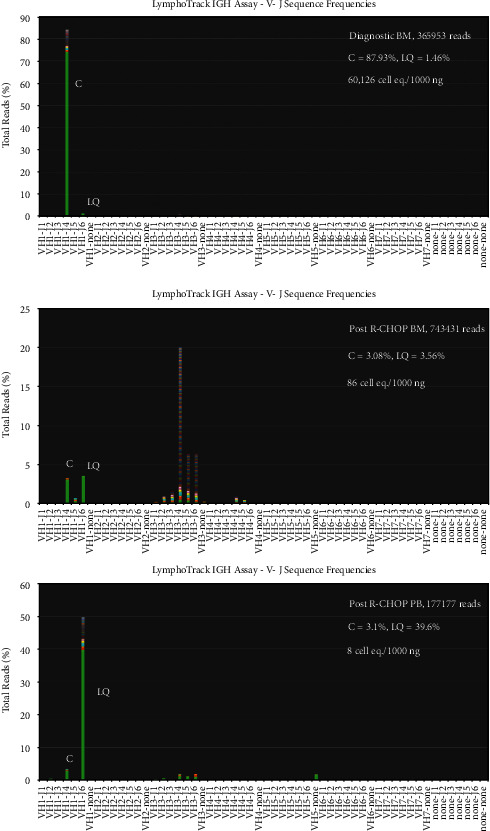
MRD after R-CHOP therapy. Clonal reads were detected at a low level in total DNA from bone marrow (BM) and peripheral blood (PB) and after R-CHOP therapy. The B-cell repertoire shows severe contraction in the peripheral blood after R-CHOP therapy. C: clonal and LQ: LymphoQuant.

**Figure 5:**
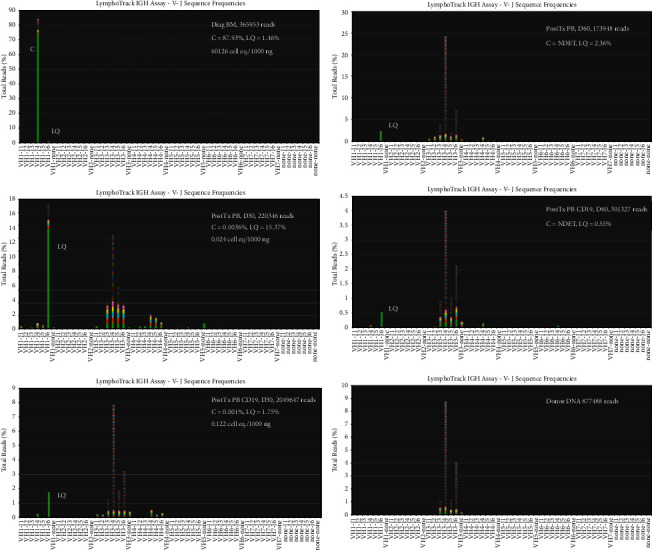
MRD after allogeneic transplant. Clonal reads were still present at day 30 after allotransplant in total DNA and CD19+ DNA but were extremely few in number to be visualized on the *Y*-axis. Clonal reads were not detected in total DNA and CD19+ B-cell DNA in peripheral blood (PB) at day 60 after transplant. The B-cell repertoire in the peripheral blood shows successful expansion and diversity at days 30 and 60 after transplant. C: clonal and LQ: LymphoQuant.

Figure 4 shows the VJ frequency graphs for the FR1-J reactions after R-CHOP therapy.  Table 2 shows the MRD data. The diagnostic bone marrow contained a tumor clone content of 60,126 cell eq./1000 ng DNA (151,515 cells). After 6 cycles of R-CHOP therapy, the tumor clone decreased by 2.84 logs to 86 cell eq./1000 ng DNA. The clonal sequence was also detected in the peripheral blood at a level of 8 cell eq./1000 ng of DNA indicating that the tumor clone was also circulating in the peripheral blood, albeit at a very low level. Six months later, the patient underwent a successful matched unrelated donor peripheral stem cell transplant. Analysis of posttransplant peripheral blood at day 30 showed that the clonal sequence was still present at an extremely low level in both total DNA and CD19+ B-cell DNA (very few clonal reads). However, at day 60 after transplant, the clonal sequence was not detected in either total or CD19+ B-cell DNA indicating successful eradication of the tumor clone (Figure 5). Furthermore, both total DNA and CD19+ B-cell DNA from the day 30 and 60 peripheral blood specimens showed complete donor engraftment without any evidence of recipient DNA by STR analysis (analytical sensitivity of 3% chimerism). After R-CHOP, there was a sharp contraction of the B-cell repertoire that expanded successfully after transplantation. The patient remains in remission more than two years after transplant with an excellent performance status.

## 3. Discussion

Our patient presented with DLBCL in a cervical lymph node which proved to be Richter's transformation of a previously undiagnosed CLL/SLL. Richter's transformation of CLL/SLL is an uncommon occurrence with an annual incidence of 0.5–1.0% per year and a median latency of 1.8–1.9 years from the time of diagnosis of CLL/SLL [9, 10]. Prognosis is generally very poor with a median overall survival of approximately one year [11]. Concurrent diagnosis of DLBCL and CLL/SLL, as in the present case, is rare but may be associated with improved survival compared to patients with sequential presentations. A retrospective study of patients with histologically proven Richter's transformation diagnosed between 1993 and 2018 at the Mayo clinic demonstrated that the median overall survival of patients with no prior treatment and concurrent diagnosis of CLL/SLL and Richter's transformation was 66.9 months compared to 29.4 months for patients with sequential presentations [11]. However, clonality analysis was successfully completed in less than 5% of patients in this study. Other investigators have shown that patients with CLL/SLL who develop clonally related DLBCL have a much poorer prognosis than patients who develop clonally unrelated DLBCL [12, 13]. Rossi et al. reported that clonally related Richter's transformation was associated with a median survival of 14.2 months vs. 62.5 months for clonally unrelated Richter's transformation, underscoring the importance of definitively establishing clonal status in patients with suspected Richter's transformation [13].

Richter's transformation is well known for dramatic clinical presentations with rapid tumor growth, intense constitutional symptoms, abnormal blood counts, and metabolic aberrations, which were conspicuously lacking in our patient. The development of Richter's transformation in the context of unrecognized subclinical CLL/SLL is also unusual, although not without precedent [14]. It is likely that our patient's transformation to DLBCL was detected at a very early stage due to the obvious site of origin in a cervical node. It is also possible that idiosyncratic features of the patient's DLBCL or host factors such as attenuated immunity or blunted proinflammatory responses may have mitigated against the manifestation of constitutional symptoms and other abnormal parameters.

NGS analysis of the nodal and marrow specimens in our patient showed that, although histologically distinct, the two lymphomas shared an identical productive VDJ rearrangement. Alignment analysis showed that our patient's clonal sequence was unmutated when compared to germline V1-2 ^*∗*^ 02 indicating lack of somatic hypermutation and an origin from a pregerminal center B cell that had not yet experienced the germinal center reaction. Furthermore, the patient's tumor also contained trisomy 12 by cytogenetic analysis. Unmutated V gene and trisomy 12 are associated with worse prognosis in CLL/SLL [15]. We next determined if the HCDR3 was stereotypic because BCR stereotypy has been reported to influence prognosis in CLL/SLL [16, 17]. Stereotypy is a feature whereby different patients with CLL have the same VDJ rearrangement with nearly identical HCDR3 sequences. Stereotypic BCRs have been identified in separate patients with CLL even though the random chance of this event occurring in different patients is almost impossible given the number of *V*, *D*, and *J* segments and the random additions and deletions of nucleotides that occur at the *V*-D and D-J junctions during VDJ rearrangement. Healthy persons also seem to carry stereotypic clones that can potentially transform into CLL/SLL [16]. In a study that included the analysis of 7596 IGH rearrangements in CLL/SLL, 18% of HCDR3 sequences were stereotypic [6]. Along with unmutated *V* genes, BCR stereotypy is associated with higher risk of Richter's transformation [15]. In particular, subsets #1, #2, and #8 stereotyped BCRs have the highest risk of transformation to large B-cell lymphoma, whereas subset #4 generally runs a more indolent course [7, 18]. Our patient's HCDR3 sequence was heterogeneous and did not fit into any of the described 19 stereotypic subsets. Because additional subsets are being defined, it is possible that our patient's HCDR3 may belong to a subset that is not yet well characterized at present. In the future, along with mutational status of the *V* gene, stereotypy might better refine prognosis in CLL/SLL patients.

We quantitated the minimal residual disease in our patient by NGS using the clonal VDJ sequence that was identified in the bone marrow specimen. In general, minimal residual disease in clonal B-cell proliferations can be detected by three methods, clone-specific PCR and detection by gel/capillary electrophoresis or real-time PCR, multiparameter flow cytometry, or NGS. For clone-specific PCR, the clonal VDJ rearrangement has to be Sanger sequenced initially. Once the sequence of the VDJ rearrangement is known, PCR primers specific to the clonal VDJ rearrangement must be designed and optimized. Because each patient's clone contains a different sequence, a PCR primer set specific to that clone must be designed for each patient. As such, this method is rather cumbersome and laborious and creates logistical problems with record keeping [19]. If the clonal sequence mutates in the primer-binding regions during follow-up, then false negative results may occur. Multiparameter flow cytometry is a sensitive method to track the malignant clone based on expression of CD markers on the surface of neoplastic B cells [20]. However, this technique requires expensive equipment and a significant level of expertise in interpreting results [19]. Therapy-induced changes in phenotypic expression in the neoplastic clone might lead to interpretation problems. Moreover, the sensitivity of detection may be less than that of PCR-based methods, especially if the assay is not fully optimized or if cellular integrity is compromised. NGS-based analysis of MRD is relatively straightforward in that the same assay that was used at diagnosis can be used during follow-up without the need to design clone specific primers. Compared to the other two methods mentioned above, the technique has superior analytical sensitivity and can detect at least 10^−6^ tumor cells if an adequate amount of DNA is analyzed. NGS also uses equipment that is available in most clinical laboratories and can shed light on the composition of the B-cell repertoire that is not possible with the previous two methods. Moreover, a significant advantage of our assay is that the software for bioinformatic analysis is included and as such, analysis of NGS data is relatively straightforward. The assay can be done in-house with access to the raw data as opposed to sending samples out for analysis to another lab and receiving only a report. Access to raw data allows assessment of quality metrics and detailed analysis of VDJ rearrangements including CDR3 sequences as detailed in this report.

A notable aspect of our NGS assay was the ability to quantitate the tumor clone in cell equivalents/input DNA using a LymphoQuant control that was added to the initial PCR step. The LymphoQuant control contains DNA from a specified amount of tumor cells mixed with polyclonal DNA. It also serves as a process control for the entire workflow. After R-CHOP therapy, the tumor clone decreased by 2.84 logs in the bone marrow when compared to the diagnostic specimen. Even though PET-CT scan showed resolution of lymph node disease, circulating tumor cells were still detected by NGS at a very low level in the peripheral blood after R-CHOP (8 clonal cell equivalents/1000 ng DNA) therapy with a potential for relapse at a later time. Interestingly, the clonal sequence was not detected in the bone marrow or peripheral blood after allogeneic transplantation at day 60 in both total DNA as well as in CD19+ B-cell DNA. These results suggest that R-CHOP alone was insufficient to eliminate the tumor clone and that allogeneic transplant was necessary for deep molecular remission. Our results show that the exquisite sensitivity and ability to track the clonal sequence make NGS the method of choice for MRD detection in lymphoma patients. MRD analysis may have utility in predicting response to therapy or risk of relapse and as such may help to optimize patient management by undertaking preemptive measures. Analyzing CD19+ B-cell DNA increases the sensitivity of detecting the B-cell tumor clone akin to lineage specific chimerism after transplant for leukemias and is required for optimal sensitivity. However, the clinically useful limits of MRD that are associated with prognosis or therapy response are unknown at the present time. More studies are needed to define the kinetics of NGS-based MRD detection in lymphoma, and it remains to be seen whether a deep molecular remission predicts long-term prognosis.

In summary, we show the utility of NGS-based clonality testing for in-depth analysis of B-cell lymphomas. First, we established the clonal relationship between the two lymphomas in our patient. Because NGS analysis provided us with the sequence and not just the length of the clonal VDJ rearrangement, we were able to determine that both lesions contained the same B-cell clone, despite having different histologies. As such, we confirmed that the DLBCL in the lymph node was due to Richter's transformation. At the same time, sequence analysis showed that the clone lacked SHM, a prognostic marker in CLL/SLL. NGS-based sequence analysis simultaneously allowed us to determine if the HCDR3 of the patient's clone belonged to a stereotypic subset. The ability to determine stereotypy is advantageous because different subsets are associated with variable prognosis. Our patient's HCDR3 however did not belong to any of the currently well-characterized subsets. As more subsets are characterized, it may be possible to use stereotypic analysis to refine prognosis. Finally, compared to flow cytometry or capillary electrophoresis, NGS analysis allowed us to monitor minimal residual disease in follow-up specimens with extremely high sensitivity and specificity. Our report illustrates that the exponential amount of information obtained by NGS can be harnessed not only to determine clonal relationships but also to refine prognosis and assess minimal residual disease with implications for prognosis and response to therapy.

## Figures and Tables

**Table 1:** Identification of a clonal population in the lymph node and bone marrow.

	Total reads	Clonal (%)	LQ (%)	PCB (%)
*Lymph node*				
FR1-J	29,955	41.81	40.96	0.92
FR2-J	83,646	47.94	26.23	0.25
FR3-J	241,759	81.25	12.56	0.43

*Diagnostic bone marrow*				
FR1-J	365,953	87.83	1.46	0.07
FR2-J	183,447	67.18	1.83	0.40
FR3-J	292,806	82.76	2.24	0.42

Total reads, % clonal, % LymphoQuant (LQ), and % polyclonal background (PCB) reads from FR1-J, FR2-J, and FR3-J primer sets are shown. The clonal VDJ rearrangement was V1-2^*∗*^02/*D*5-12^*∗*^01/J4^*∗*^02. The LQ rearrangement was V1-69^*∗*^01/*D*6-13^*∗*^01/J6^*∗*^03.

**Table 2:** Minimal residual disease detection by NGS.

Specimen	Input DNA (ng)	Total reads	Reads				Clonal cell eq. per input DNA	Clonal cell eq. per 1000 ng	Tumor clone, interpretation
			Clonal	LQ	(%) clonal	(%) LQ			
Diagnostic BM	100	365953	321793	5352	87.93	1.46	6013	60126	Detected
After R-CHOP BM	1000	743431	22865	26454	3.08	3.56	86	86	Detected
After R-CHOP PB	1000	177177	5487	70158	3.10	39.60	8	8	Detected
Post Tx PB, TDNA, day 30	1000	220346	8	33874	0.0036	15.37	0.024	0.024	Detected
After Tx PB, CD19+ DNA, day 30	500	2049647	22	35956	0.001	1.754	0.061	0.122	Detected
After Tx PB, TDNA, day 60	250	173948	0	4099	0	2.28	0	0	Not detected
After Tx PB, CD19+ DNA, day 60	250	301327	0	1652	0	0.55	0	0	Not detected

DNA input, total reads, % clonal or LQ reads, and clonal cell equivalents/1000 ng of DNA are shown. BM: bone marrow, PB: peripheral blood, TDNA: total DNA, CD19+: DNA from isolated CD19+ B cells, and Tx: allotransplant.

## Data Availability

Data used to support the findings of the study are included within the case report and are available upon request.

## Supplementary Materials

Supplementary Materials File 1: materials and methods. File 2: histopathology of the lymph node. .
