# Supplementary material for: NGS Analysis of Clonality and Minimal Residual Disease in a Patient with Concurrent Richter's Transformation and CLL/SLL
Source: Case Rep Hematol. 2021 Dec 28;2021:9740281. doi: 10.1155/2021/9740281 (PMC8727142; doi:10.1155/2021/9740281)
Supplement: Supplementary Materials — File 1: materials and methods. File 2: histopathology of the lymph node. . [file 9740281.f1.zip › 9740281.f1/File 1 - Materials and Methods.docx]

**SUPPLEMENTARY MATERIAL**

**MATERIALS AND METHODS**

**DNA extraction:**

DNA was extracted from FFPE sections of the lymph node by the QIAamp DNA FFPE Tissue Kit (Qiagen, cat no. 56404, Germantown, MD) according to the manufacturer’s protocol. Five sections (5µm thick) were cut and placed on glass slides. The sections were deparaffinized in xylene and followed by washing in 100% ethanol. After air drying, sections were scrapped off with a sterile blade into an Eppendorf tube containing 190µl of Buffer ATL with 10µl of Proteinase K (Roche, Cat. No. 03 115 828 001). 100µl of mineral oil was layered on the top to prevent evaporation. The sections were incubated at 56^o^C for 16 hours in a dry incubator to digest completely and then at 90^o^C for 1 hour. 2µl of RNase was added to the lysate and incubated for 5 mins to digest RNA. 200µl of Buffer AL and 200µl of absolute ethanol was added to the lysate and pipetted to mix well. The entire solution was then applied to a QIAamp MinElute column. The column was centrifuged at 6000g for 1 minute and the flow-through was discarded. The column was then washed with 500µl of buffer AW1 and buffer AW2. Finally DNA was eluted with 30µl of buffer ATE. DNA concentration and purity were measured with the Qubit fluorometer using the dsDNA HS assay kit (Thermofisher, Cat no. Q32851) and the Nanodrop spectrophotometer.

For bone marrow and peripheral blood specimens, after RBC lysis (Qiagen cat no.158922), total DNA was extracted from the leukocyte pellet by the Puregene DNA extraction kit (Qiagen, Cat no 158389) according to the manufacturer’s protocol. Either 3ml of bone marrow or 5ml of peripheral blood was used for DNA extraction. CD19+ B cells were isolated from 10ml of post-transplant peripheral blood using the EasySep™ Human CD19 Positive Selection Kit II according to the manufacturer’s manual protocol (Cat no.17854, STEMCELL technologies, Vancouver, Canada). At the final step of the procedure, CD19+ B cells bound to antibody coated magnetic particles form a pellet at the bottom of the isolation tube. Lysis buffer from the Puregene DNA extraction kit DNA was added directly to the pellet and genomic DNA was extracted according to the manufacturer’s protocol. The quantity and quality of DNA was measured using a Nanodrop spectrophotometer.

**Post-transplant chimerism analysis, peripheral blood**:

Donor, pre-transplant recipient and post-transplant DNA specimens were analyzed by STR analysis using the Powerplex fusion kit according to the manufacturer’s protocol (Cat no. DC2402, 24 loci, Promega, Madison WI). First, 2ng of genomic DNA was amplified in a multiplex PCR reaction. PCR products were separated by capillary electrophoresis on an ABI3500XL instrument and data was analyzed by Genemapper 5.0 software. Recipient specific informative loci were selected and the average percent recipient DNA in the post-transplant specimen was calculated based on the area under the peaks.

**IgH clonality by NGS**:

IgH clonality was analyzed by NGS with the Lymphotrack IgH FR1/FR2/FR3 assay according to the manufacturer’s protocol (Cat no. 7-121-0139, Invivoscribe Inc, San Diego, CA). The major steps of the protocol were:

**PCR Amplification:**

First, genomic DNA from the lymph node and diagnostic bone marrow was PCR amplified with FR1/FR2/FR3 and J primers to identify clonality and to confirm the clonal relationship between the two specimen types. Prior to amplification, 2µl of LymphoQuant B cell internal control DNA (LQ, Cat no. 7-121-0139, 50 cell equivalents/µl, Invivoscribe) was added to each DNA specimen as a process control. Meticulous attention was followed to avoid contamination.

For MRD analysis, DNA from the follow up specimens was amplified with FR1 and J primers only. 2µl of the LQ control (100 cell equivalents) was added to each specimen to help facilitate MRD quantitation. The follow up specimens included bone marrow and peripheral blood DNA after R-CHOP therapy and peripheral blood after allo-transplant at days 30 and 60. For the post-transplant peripheral blood specimens, total DNA as well as DNA from isolated CD19+ B cells were analyzed. Donor DNA was also amplified with FR1 and J primers without the addition of the LQ control.

**Library preparation and sequencing**:

After amplification, PCR products were purified and the libraries were quantitated by the Kappa Library quantiation kit according to the manufacturer’s protocol (Roche, Cat no. KK4824, Pleasanton, CA). Length corrected library concentrations were calculated using a 1:4000 to 1:100,000 dilution of the PCR products. Next, separate FR1, FR2 and FR3 libraries were prepared. Each library contained approximately 4nM concentration of amplicon from each sample. After another round of quantitation, FR1, FR2 and FR3 libraries were combined in a total volume of 18µl according to the manufacturer’s instructions. 2ul of freshly prepared 1N NaOH was added to denture DNA (pooled library concentration = 2nM). After 5 minutes at room temperature, 980 ul of chilled HT1 buffer was added to a volume of 1000µl (pooled library concentration = 40pM). A further dilution was made by adding 300µl of denatured and diluted library from the previous step to 700µl of HT1 buffer (pooled library final concentration = 12pM). 600µl of the final dilution was loaded into a Miseq v2 reagent cartridge (300 cycles) and run on a Miseq instrument with paired end sequencing. Each Miseq run took about 40 hours. Adapter trimming was specified in the sample sheet.

**Data analysis**:

Overall metrics for each Miseq run were visualized in Sequence Analysis Viewer v2.4.5 (Illumina). All runs had adequate quality metrics in both R1 and R2 reads: Q>30 in the first 200 cycles and >20 in subsequent cycles. The prephasing and phasing rates were very low (<0.2). In general, about 6 – 8 Gb of data was generated with a cluster density of around 600K/mm. >90% of reads had a score of >Q30. Next, each fastq.gz file was analyzed individually for quality metrics by the FastQC program (Babraham Bioinformatics). Upto cycle 200, all fastq files contained reads with a median score of >Q30 (first and 3^rd^ quartiles 25 and 40). Beyond cycle 200, the median Q score was about 25. >95% of reads had a sequence length of 250nt except in FFPE derived DNA specimen from the lymph node. In this specimen, a portion of the reads were between 105 – 155bp. This is to be expected because of fragmented DNA in FFPE specimens. Nevertheless, sufficient high quality reads were generated by FR1-J and FR2-J primer sets even in FFPE derived DNA from the lymph node.

IgH clonality analysis was performed by importing the fastq.gz files into Lymphotrack software v2.4.3 (Invivoscribe). This analysis defined the clonal sequence each specimen for FR1, FR2 and FR3 reactions. The Lymphotrack software generates a list of the top 200 most frequently occurring sequences (read summary) and then combines the reads from sequences with the same V and J assignment if they differ by 1-2 nt to create a separate merged read summary. Using the merged read summary, a clonal sequence was defined as a sequence with a read frequency of >2.5% and a frequency of >10X the background (generally the 3^rd^ sequence in the merged read summary)^4^.

**Detailed analysis of the clonal sequence (FR1-J)**:

V, D and J assignments, % similarity to germline V, junctional and HCDR3 features and whether the rearrangement was productive/unproductive were characterized by the web based IMGT/V-Quest and Ig-BLAST programs (<http://www.imgt.org/IMGT_vquest/vquest>; <https://www.ncbi.nlm.nih.gov/igblast/>). In addition, the clonal sequence was also aligned to the germ line V gene to determine the presence of somatic hypermutation using the Clustal Omega program (<https://www.ebi.ac.uk/Tools/msa/clustalo/>). HCDR3 subset analysis was performed with the ARRest/AssignSubsets program (<http://tools.bat.infspire.org/arrest/assignsubsets/>).

**MRD analysis using the FR1-J sequence:**

For MRD analysis, each sample was spiked with 2µl of LymphoQuant control (~100 clonal cell equivalents) before PCR amplification with FR1 and J primers. Initially, the fastq.gz files were analyzed by the Lymphotrack v2.4.3 software. Among many outputs, the software generated a unique reads file. The unique reads file was imported into LymphotrackMRD software v1.2.0 (Invivoscribe) to determine MRD. This latter software searches the unique reads file for the designated clonal and LQ sequences. For each sample, the % clonal and % LQ sequence were calculated as follows:

(Exact match clonal sequence reads / Total reads) x 100 = % clonal sequence

(LQ clonal sequence reads / Total reads) x 100 = % LQ clonal sequence

MRD in cell equivalents/DNA input (ng) was calculated as follows:

MRD = (% clonal sequence / % LQ clonal sequence) x 100 cell equivalents

**Controls**:

Clonal and polyclonal DNA controls along with a no-template control (water) were included in each run. Clonal and polyclonal control DNA yielded expected results with an adequate number of reads. The total number of reads in a representative clonal control were 824,481 in FR1, 344,865 in FR2 and 594,251 in FR3 reactions. The topmost sequence had a read frequency of 5.69% in FR1, 8.51% in FR2 and 9.11% in FR3. IMGT V-Quest and IgBLAST analysis of the FR1-J sequence showed that the rearrangement was V1-46*01/D2-2*01/J4*02 with the V region being un-mutated when compared to germline sequence. In a representative polyclonal control, total number of reads were 920,623 in FR1, 325,225 in FR2 and 394,464 in FR3. The topmost sequences had read frequencies of 0.1%, 0.05% and 0.09% in the three reactions respectively.
