# Supplementary figures and images for: NGS Analysis of Clonality and Minimal Residual Disease in a Patient with Concurrent Richter's Transformation and CLL/SLL
Source: Case Rep Hematol. 2021 Dec 28;2021:9740281. doi: 10.1155/2021/9740281 (PMC8727142; doi:10.1155/2021/9740281)

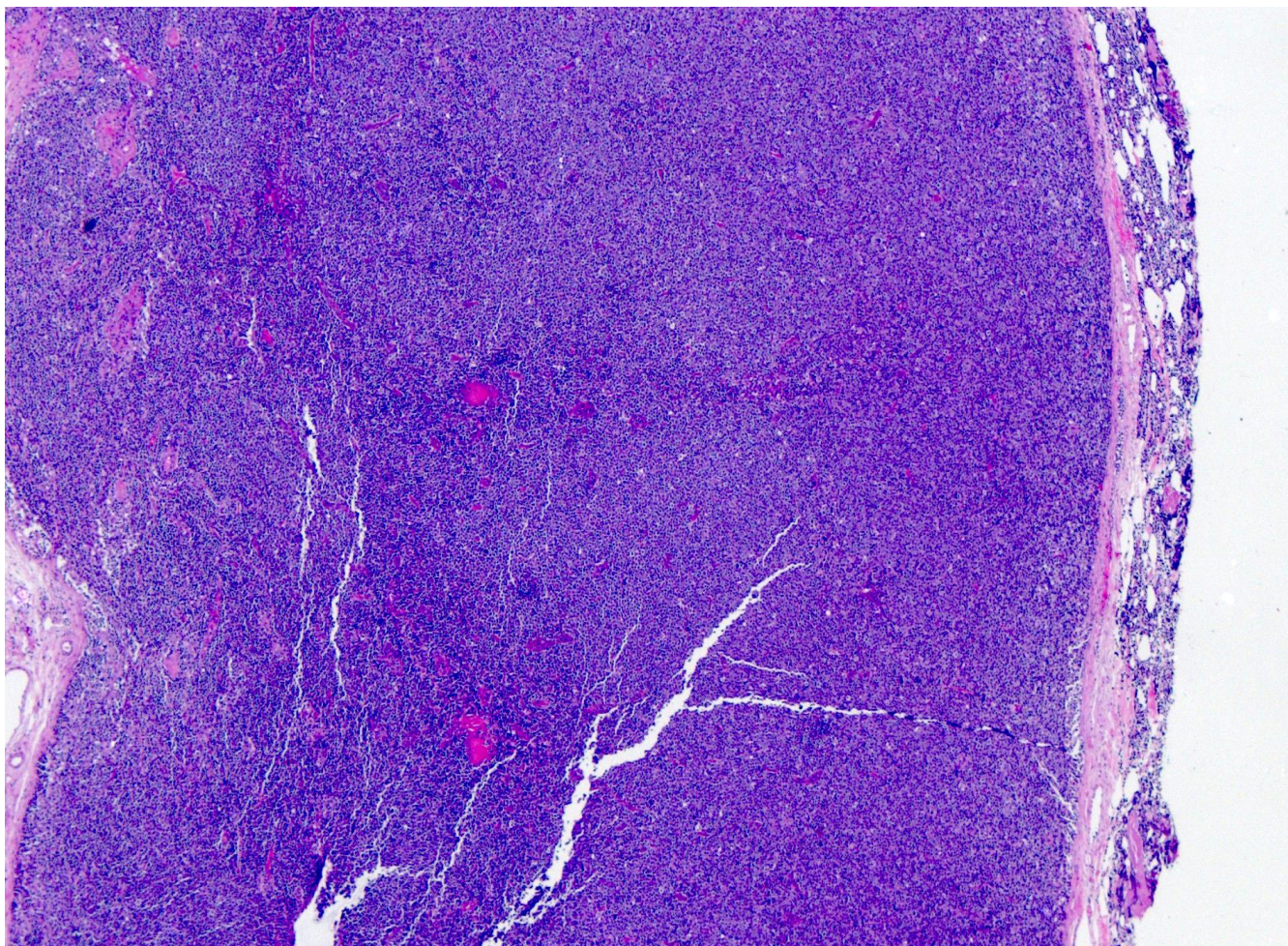

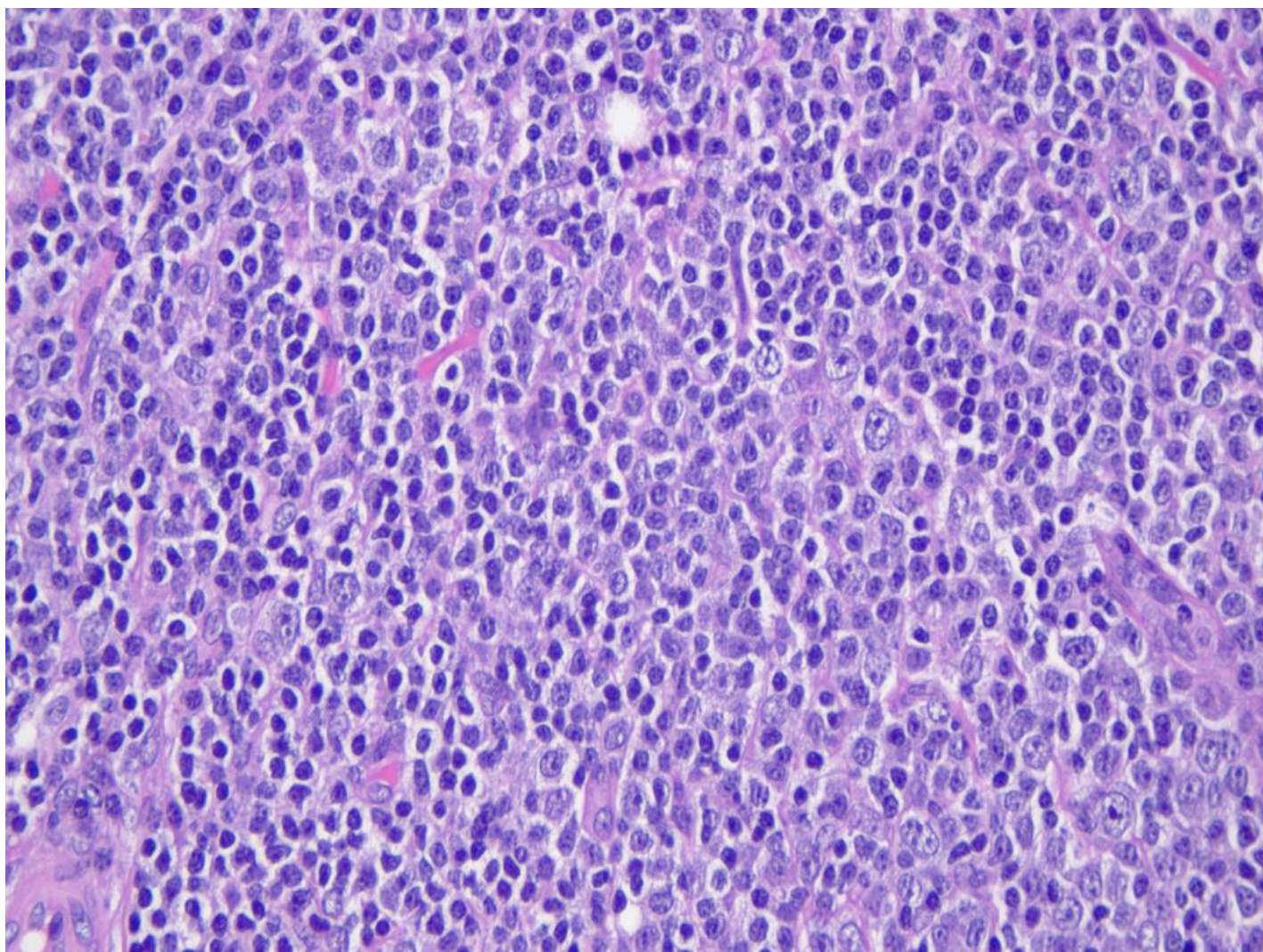

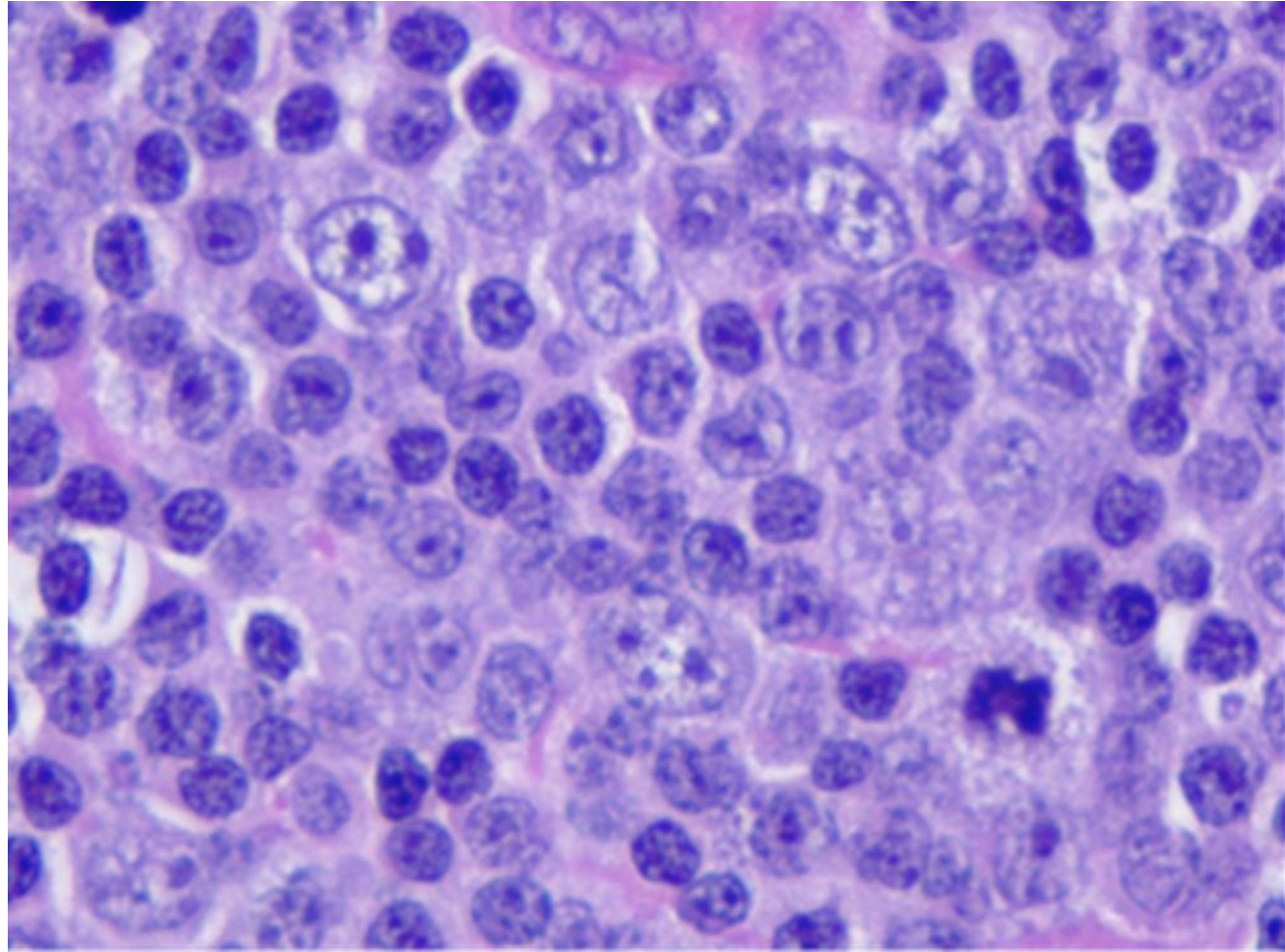

Supplement: Supplementary Materials — File 1: materials and methods. File 2: histopathology of the lymph node. . [file 9740281.f1.zip › 9740281.f1/File 2 - Histopathology Lymphnode.pdf]
